# A Universal New Definition of Heart Failure With Improved Ejection Fraction for Patients With Coronary Artery Disease

**DOI:** 10.3389/fphys.2021.770650

**Published:** 2021-12-03

**Authors:** Haozhang Huang, Jin Liu, Min Lei, Zhou Yang, Kunming Bao, Qiang Li, Wenguang Lai, Bo Wang, Yibo He, Shiqun Chen, Chun-Quan Ou, Maimaitiaili Abudukerimu, Yuying Hu, Ning Tan, Jiyan Chen, Yong Liu

**Affiliations:** ^1^Guangdong Provincial Key Laboratory of Coronary Heart Disease Prevention, Department of Cardiology, Guangdong Provincial People’s Hospital, Guangdong Cardiovascular Institute, Guangdong Academy of Medical Sciences, Guangzhou, China; ^2^The Second School of Clinical Medicine, Southern Medical University, Guangzhou, China; ^3^Department of Ultrasound, Fuwai Yunnan Cardiovascular Hospital, Kunming, China; ^4^State Key Laboratory of Organ Failure Research, Guangdong Provincial Key Laboratory of Tropical Disease Research, Department of Biostatistics, School of Public Health, Southern Medical University, Guangzhou, China; ^5^Department of Cardiology, Longyan First Affiliated Hospital of Fujian Medical University, Longyan, China; ^6^School of Biology and Biological Engineering, Guangdong Provincial People’s Hospital, South China University of Technology, Guangzhou, China; ^7^Department of Cardiology, First People’s Hospital of Kashgar, Kashgar, China; ^8^School of Medicine, Guangdong Provincial People’s Hospital, South China University of Technology, Guangzhou, China

**Keywords:** heart failure with improved ejection fraction, coronary artery disease, left ventricular ejection fraction, characteristic, mortality, heart failure

## Abstract

**Aims:** The aims of this study were to describe the characteristics and outcomes of the universal new definition of heart failure with improved ejection fraction (HFimpEF) and to identify predictors for HFimpEF among patients with coronary artery disease (CAD).

**Methods:** CAD subjects with heart failure with reduced ejection fraction (HFrEF) (EF ≤ 40%) at baseline were enrolled from the real-world registry of the Cardiorenal ImprovemeNt study from January 2007 to December 2018. The new definition of HFimpEF was defined as left ventricular EF (LVEF) of≤40% at baseline and with improvement of up to 40% and at least a ≥ 10% increase during 1 month to 1 year after discharge.

**Results:** Of the 747 CAD patients with HFrEF (86.7% males, mean age: 61.4 ± 11 years), 267 (35.7%) patients conformed to the new HFimpEF definition. Patients with HFimpEF were younger (adjusted odds ratio [aOR]: 0.98 [0.97–0.99]) and had a higher rate of hypertension (aOR:1.43 [1.04–1.98]), lower rate of percutaneous coronary intervention (PCI) treatment at the time of detection of HFrEF (aOR: 0.48 [0.34–0.69]), history of PCI (aOR: 0.51 [0.28–0.88]), history of acute myocardial infarction (aOR: 0.40 [0.21–0.70]), and lower left ventricular end diastolic diameter (aOR: 0.92 [0.90–0.95]). During 3.3-year follow-up, patients with HFimpEF demonstrated lower rates of long-term all-cause mortality (13.1% vs. 20.8%, aHR: 0.61[0.41–0.90]).

**Conclusion:** In our study, CAD patients with HFimpEF achieved a better prognosis compared to those with persistent HFrEF. Patients with CAD meeting the criteria for the universal definition of HFimpEF tended to be younger, presented fewer clinical comorbidities, and had lower left ventricular end diastolic diameter.

## Introduction

Epidemiological data further demonstrate that heart failure (HF) is a global disease with increasing prevalence and burden (Bozkurt et al., 2021). The most common cause of HF is coronary artery disease (CAD) (Conrad et al., 2018). Thus, it is extremely important for a physician to use important indexes to evaluate both the measure and efficacy of therapy outcome among CAD patients with HF.

Recently, a scientific panel has proposed a new working definition of HF with improved ejection fraction (HFimpEF) that includes a baseline left ventricular ejection fraction (LVEF) of ≤ 40%, a ≥ 10% increase from baseline LVEF, and a second measurement of LVEF of>40% (Wilcox et al., 2020; Bozkurt et al., 2021). The most recent consensus indicated the necessity to employ and use a universal definition and classification in research studies. In consequence, differences in the prognosis and clinical management of new patients with HFimpEF and those without improvement have attracted increasing attention, especially among patients with CAD.

However, current studies on the prognosis based on the new definition of HFimpEF are lacking. Thus, we sought to comprehensively investigate the clinical characteristics and outcomes of HFimpEF among patients with CAD.

## Materials and Methods

### Data Collection

This single-center, retrospective, and observational study was based on the registry of Cardiorenal ImprovemeNt (CIN, ClinicalTrials.gov NCT04407936) cohort from January 2007 to December 2018 at the largest cardiovascular center in south China, the Guangdong Provincial People Hospital (GDPH). In patients experiencing CAD, according to the diagnosis of the 10th Revision Codes of the International Classification of Diseases (ICD-10; I20.xx–I25.xx, I50.00001, and I91.40001), percutaneous coronary intervention (PCI) or coronary angiography (CAG) was performed in compliance with standard clinical practice guidelines (Jneid et al., 2012; Levine et al., 2016). The baseline information was as follows: demographics, laboratory test results, procedures, and other clinical variables. Blood samples were collected in the early morning after overnight fasting.

### Study Population

**FIGURE 1:**
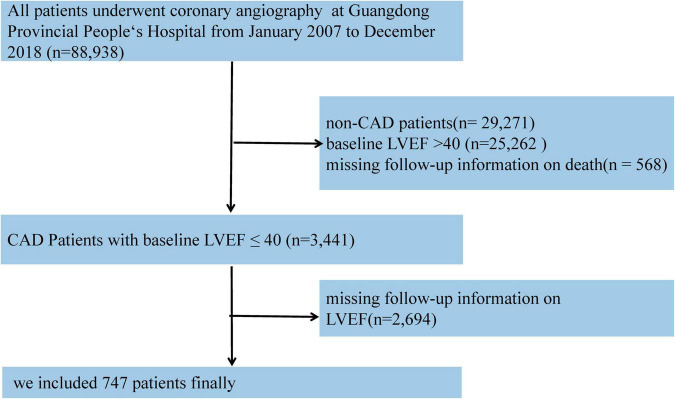
The flow of participants through the trial.

Based on the CIN database, CAD patients with LVEF ≤ 40% in admission and at least one echocardiogram re-examination during the 1-month to 1-year follow-up were enrolled in our study. If patients had undergone several echocardiogram examinations over time, we used the latest examination during the follow-up echocardiogram to classify EF improvement. Those without baseline data relative to LVEF and death outcomes during follow-up were excluded. Eventually, 747 patients were included in the study (Figure 1).

### Definition and End Points

The LVEF measurements were performed by a senior echocardiography physician at the GDPH using standardized procedures, and they were responsible for the data quality control and periodical data verification. During the 1-year follow-up, the patient was invited to return to the hospital for LVEF re-examination by telephone.

Heart failure status was assessed according to signs, symptoms, and guideline-based laboratory tests (Ponikowski et al., 2016; Bozkurt et al., 2021). The HFimpEF was defined as a baseline LVEF value of ≤ 40%, a ≥ 10% increase from baseline LVEF, and a second measurement of LVEF of > 40%. Chronic kidney disease (CKD) was defined as an estimated glomerular filtration rate (eGFR) < 60 ml/min/1.73 m^2^. eGFR was calculated using the Modification of Diet in Renal Disease (MDRD) equation (Aguiar-Souto et al., 2010).

The primary end point of this study was long-term all-cause death. Data on all-cause death and follow-up time were obtained from the Guangdong Provincial Public Security and matched to the electronic Clinical Management System of the Guangdong Provincial People’s Hospital records.

### Statistical Analysis

Descriptive statistics are reported as the mean [standard deviation (SD)], median [interquartile range (IQR)] or number and percentage when appropriate. Differences between the two groups were analyzed using Student’s *t*-test. Missing values in the candidate predictor variables were imputed by multivariate imputation using the method of the chained equation with missing-at-random assumptions, to avoid exclusion of patients with missing values. Patients with missing predictors>20% were excluded from the model derivation (Liao et al., 2014).

**FIGURE 2:**
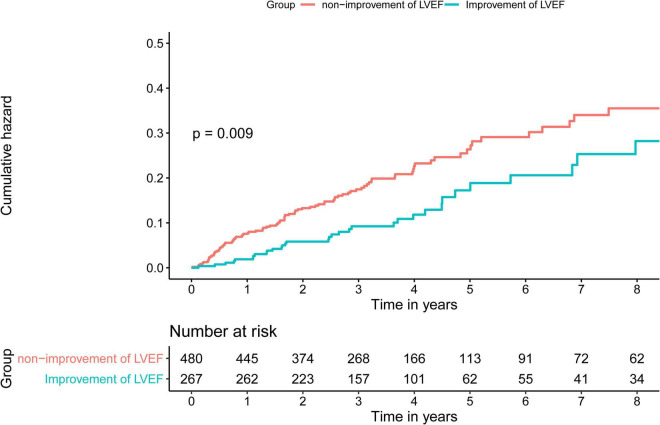
Kaplan-Meier curves for long-term all-cause mortality of heart failure with reduced ejection fraction (HFrEF) among patients with coronary artery disease (CAD).

**FIGURE 3:**
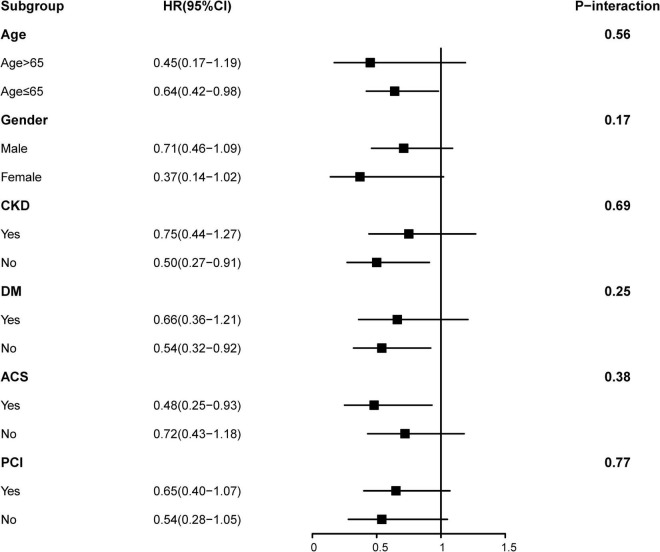
Hazard ratios for the all-cause mortality in different subgroups (heart failure with improved ejection fraction (HFimpEF) vs. persistent HFrEF).

Time-to-event data are presented graphically using Kaplan-Meier curves (Figure 2). Log-rank tests were used to compare survival between the two groups. The association between HFimpEF and the study end points was assessed by the multivariate Cox analyses using different models. Hazard ratios (HRs) and 95% confidence intervals (CIs) were calculated. Model 1 was unadjusted, model 2 was adjusted for age (as a continuous variable) and sex, and model 3 included variables that were associated with mortality according to clinical experience and previous literature (Perry et al., 2020) (including Model 1 and 2 variables, anemia, CKD, diabetes mellitus, acute myocardial infarction (AMI), PCI, chronic obstructive pulmonary disease, atrial fibrillation, and baseline LVEF). Finally, the results of Model 3 were defined as the primary results. We also performed subgroup analysis among four prespecified subgroups to assess the HFimpEF on all-cause mortality [older age (>65 or≤65 years), sex (male or female), diabetes mellitus (yes or no), and CKD (yes or no)] (Figure 3).

To evaluate characteristics associated with HFimpEF among CAD patients with baseline LVEF ≤ 40%, we used logistic regression. Clinically indicated variables and all variables with statistically significant differences between the two groups in Table 1 were considered as candidate variables, and backward stepwise variables selection was used to derive the final model,

**TABLE 1:** Baseline characteristics of the patients with persistent HFrEF or HFimpEF.

Characteristic	Overall	Persistent HFrEF	HFimpEF	*P*-value
	(*n* = 747)	(*n* = 480)	(*n* = 267)	
**Demographic characteristics**				
Female, n (%)	99 (13.3)	61 (12.7)	38 (14.2)	0.634
Age, years, mean (SD)	61.4 (11.0)	61.69 (10.79)	60.81 (11.33)	0.294
Medical insurance				0.932
Self-paying	93 (12.4)	60 (12.5)	33 (12.4)	
Urban insurance	566 (75.8)	366 (76.2)	200 (74.9)	
Rural insurance	27 (3.6)	16 (3.3)	11 (4.1)	
Other	61 (8.2)	38 (7.9)	23 (8.6)	
**Medical history**				
Anemia, n (%)	254 (35.2)	172 (36.9)	82 (32.2)	0.232
AMI, n (%)	173 (23.2)	109 (22.7)	64 (24.0)	0.763
DM, n (%)	263 (35.2)	171 (35.6)	92 (34.5)	0.81
CKD, n (%)	295 (40.7)	202 (43.3)	93 (36.0)	0.066
AF, n (%)	31 (4.1)	19 (4.0)	12 (4.5)	0.872
Stroke, n (%)	41 (5.5)	26 (5.4)	15 (5.6)	>0.99
COPD, n (%)	6 (0.8)	4 (0.8)	2 (0.7)	>0.99
Hypertension, n (%)	359 (48.1)	217 (45.2)	142 (53.2)	0.044
History of AMI, n (%)	92 (12.3)	76 (15.8)	16 (6.0)	<0.001
History of CABG, n (%)	7 (0.9)	5 (1.0)	2 (0.7)	0.999
History of prePCI, n (%)	85 (11.4)	66 (13.8)	19 (7.1)	0.009
**Procedure, n (%)**				
PCI	550 (73.6)	370 (77.1)	180 (67.4)	0.005
Primary PCI	489 (65.5)	321 (66.9)	168 (62.9)	0.003
DES	525 (70.3)	349 (72.7)	176 (65.9)	0.063
BES	17 (2.3)	12 (2.5)	5 (1.9)	0.768
CMV, ml, mean (SD)	147.6 (88.7)	147.3 (84.4)	148.1 (96.1)	0.91
**Laboratory tests**				
LDLC, mmol/L, median (IQR)	2.76 [2.26, 3.44]	2.74 [2.20, 3.48]	2.83 [2.33, 3.41]	0.311
HDLC, mmol/L, median (IQR)	0.91 [0.77, 1.08]	0.89 [0.77, 1.06]	0.93 [0.78, 1.09]	0.232
eGFR, ml/min/1.73m2 [mean (SD)]	67.7 (25.1)	66.4 (25.4)	70.0 (24.4)	0.075
HbA1c,% [mean (SD)]	6.80 (1.67)	6.82 (1.64)	6.76 (1.73)	0.734
hs-CRP, mg/dL [median (INR)]	7.36 [2.62, 20.90]	7.55 [2.46, 23.55]	6.73 [2.84, 20.25]	0.993
ALB, g/L [mean (SD)]	35.3 (4.5)	35.3 (4.7)	35.3 (4.1)	0.996
**Echocardiography and biology data dynamics**				
Baseline LVEF,% (mean (SD))	32.5 (5.9)	32.2 (6.0)	33.2 (5.5)	0.016
LVEDD, mm [mean (SD)]	60.1 (22.0)	62.5 (26.8)	57.5 (7.2)	0.003
ProBNP, ng/ml [median (IQR)]	2,396 [1,149, 5,456]	2,430 [1,147, 5,733]	2,371 [1,184, 4,778]	0.643
NYHA class dynamics				0.396
1	88 (15.6)	58 (15.8)	30 (15.3)	
2	243 (43.1)	159 (43.2)	84 (42.9)	
3	191 (33.9)	119 (32.3)	72 (36.7)	
4	42 (7.4)	32 (8.7)	10 (5.1)	
**Medications**				
ACEI/ARB, n (%)	400 (54.3)	276 (58.2)	124 (47.1)	0.005
Beta-blockers, n (%)	641 (87.0)	412 (86.9)	229 (87.1)	>0.99
CCB, n (%)	89 (12.1)	45 (9.5)	44 (16.7)	0.006
Statin, n (%)	699 (94.8)	453 (95.6)	246 (93.5)	0.307
Aspirin, n (%)	655 (88.9)	429 (90.5)	226 (85.9)	0.077
Clopidogrel, n (%)	620 (84.1)	410 (86.5)	210 (79.8)	0.024
MRA, n (%)	503 (68.2)	326 (68.8)	177 (67.3)	0.742
Discharge status, n (%)				0.604
Medical advice discharge	742 (99.3)	476 (99.2)	266 (99.6)	
Automatic withdraw	2 (0.3)	2 (0.4)	0 (0.0)	
In-hospital death	2 (0.3)	1 (0.2)	1 (0.4)	

*HFrEF, heart failure with reduced ejection fraction; HFimpEF, heart failure with improved ejection fraction; ACEI/ARB, angiotensin-converting enzyme inhibitor/angiotensin receptor blocker; AF, atrial fibrillation; ALB, albumin; AMI, acute myocardial infarction; BES, bare metal stent; CABG, coronary artery bypass grafting; CCB, calcium channel blockers; CKD, chronic kidney diseases; COPD, chronic obstructive pulmonary disease; DES, drug eluting stents; DM, diabetes mellitus; eGFR, estimated glomerular filtration rate; HbA1c, hemoglobin A1c; HDL-C, high-density lipoprotein cholesterol; hs-CRP, hypersensitive C-reactive protein; PCI, percutaneous coronary intervention; LDL-C, low-density lipoprotein cholesterol; MRA, mineralocorticoid receptor antagonist; LVEF, left ventricular ejection fraction; LVEDD, left ventricular end-diastolic dimension.*

where adjusted odds ratios (OR) were reported with 95% CIs. All *P*-values were two-sided, with *P*-values < 0.05 statistically significant. All statistical analyses were performed using R (version 4.0.3).

### Sensitivity Analysis

The sensitivity analysis was used to verify the reliability of the data filtering process and the results of multiple fill analysis under the random miss hypothesis (Supplementary Tables 1, 2; Conrad et al., 2018) we only included data from 6 months or more of LVEF follow-up and the association between HFimpEF and the study end points were assessed by multivariate Cox analyses using model 2 (Supplementary Table 3).

## Results

### Characteristics of Patients

A total of 747 patients identified from admission records were divided into two groups (persistent HFrEF vs. HFimpEF) according to the improvement of EF at the 1-year follow-up. The mean age was 61.4 ± 11.0 years, more than 80% of patients were male (*n* = 648), 173 (23.2%) presented with AMI, 92 (12.3%) had history of AMI, and 550 (73.6%) underwent PCI treatment. The individuals with improved EF at the 1-year follow-up accounted for 35.7% (*n* = 267) of patients. Compared to patients with persistent HFrEF, the mean age of patients with HFimpEF was 61.7 ± 10.8 years and males accounted for 85.8% (*n* = 229). Patients with HFimpEF presented more favorable characteristics than patients with persistent HFrEF: fewer PCI, history of AMI, history of PCI, higher percentage of use of drugs such as renin-angiotensin-aldosterone system (RAAS) inhibitors, clopidogrel, and calcium channel blockers. The distribution of echocardiography indicators among CAD patients with persistent HFrEF and HFimpEF was also significantly different, such as baseline LVEF and left ventricular end-diastolic dimension (LVEDD). While, NT-pro brain natriuretic peptide (NT-proBNP) and NYHA class dynamics did not differ significantly. Additional details of the baseline characteristics of the enrolled patients are reported in Table 1.

### Primary Outcomes

During the median 3.3 years (IQR: 2.2–4.7) long-term follow-up, 135 (18.1%) deaths occurred: 35 deaths (13.1%) in the HFimpEF group and 232 (20.8%) in the persistent HFrEF group. As determined by Kaplan-Meier curves (Figure 2), HF patients with improved EF achieved significantly lower mortality and better long-term prognosis in terms of survival in years than persistent HFrEF (log-rank *P* < 0.001). HFimpEF has a better prognosis in all three models, which was indicated by the multivariable-adjusted model 3, in which HF patients with improved EF had lower all-cause mortality risk than persistent HFrEF (full-adjusted HR 0.61; 95%: 0.4–0.90, *P* = 0.003) (Table 2).

**TABLE 2:** The association between HFrEF and mortality in different models among patients with CAD.

	Long-term all-cause mortality HR, 95% Cl, *P*-value		
	Model 1^a^	Model 2^b^	Model 3^c^
Persistent HFrEF	Ref	Ref	Ref
HFimpEF	0.60 (0.41–0.88), 0.010	0.60 (0.40–0.87), 0.008	0.61 (0.41–0.90), 0.003

*^a^Unadjusted.*

*^b^Adjusted age and gender.*

*^c^Adjusted age, gender, anemia, chronic kidney disease, diabetes mellitus, acute myocardial infarction, percutaneous coronary intervention, chronic obstructive pulmonary disease, atrial fibrillation, baseline LVEF.*

*CAD, coronary artery disease; HR, hazard ratio.*

### Predictors of Heart Failure With Improved Ejection Fraction and Model Construction

We investigated independent predictors of patients with HFimpEF (C-index = 0.71). In the multivariable analysis, hypertension (aOR: 1.43, 95% CI: 1.04–1.98) was associated with an increased likelihood of HFimpEF. While the increase in age (aOR: 0.98; 95% CI: 0.97–0.99), PCIs (aOR: 0.48; 95% CI: 0.34–0.69), history of PCI (aOR: 0.51; 95% CI: 0.28–0.88), history of AMI (aOR: 0.40; 95% CI: 0.21–0.70), and increase of left ventricular end diastolic diameter (aOR: 0.92; 95% CI: 0.90–0.95) were associated with decreased likelihood of HFimpEF (Table 3).

**TABLE 3:** Univariate and multivariate analysis with backward stepwise of baseline patient characteristics associated with HFimpEF (vs. persistent HFrEF).

Predictors for HFimpEF among baseline LVEF ≤ 40		
	OR(95% Cl)	aOR(95% Cl)*
Age, per 1 year increase	0.99 (0.98–1.01)	0.98 (0.97–0.99)
Female (vs. male)	1.14 (0.73–1.76)	
CKD (vs. non-CKD)	0.73 (0.54–0.99)	0.75 (0.54–1.05)
AF (vs. non-AF)	1.14 (0.53–2.36)	
COPD (vs. non-COPD)	0.90 (0.12–4.63)	
PCI (vs. non-PCI)	0.62 (0.44–0.86)	0.48 (0.34–0.69)
History of AMI (yes vs. no)	0.34 (0.19–0.58)	0.40 (0.21–0.70)
History of PCI (yes vs. no)	0.48 (0.27–0.80)	0.51 (0.28–0.88)
Hypertension	1.38 (1.02–1.86)	1.43 (1.04–1.98)
Baseline ejection fraction, per 1% increase	1.03 (1.01–1.06)	
Left ventricular end diastolic diameter, per 1 mm increase	0.93 (0.91–0.95)	0.92 (0.90–0.95)
Final Model C-index = 0.71		

*Backward stepwise logistic regression.

*CKD, chronic kidney diseases; AF, atrial fibrillation; COPD, chronic obstructive pulmonary disease; AMI, acute myocardial infarction; PCI, percutaneous coronary intervention.*

### Subgroup Analysis

In Figure 3, the subgroup analysis of results on some groups showed mixed results. Positive associations that had previously been observed in the overall population were seen in non-older, non-CKD, non-DM, and ACS subgroups. However, all two-way interactions remained non-significant (all *P* > 0.1). We speculated that HFimpEF was associated with consistent risk of mortality across dichotomized subgroups (older age [>65 or≤65 years], sex [male or female], diabetes mellitus [yes or no], and CKD [yes or no]). Furthermore, no effect was found in PCI [yes or no]) and ACS group [yes or no] (*P* for interaction > 0.05). Importantly, our correlation results need to be interpreted with caution (Figure 3).

## Discussion

This is the first study to investigate the characteristics and outcomes of a universal new definition of HFimpEF among patients with CAD. Our study showed that CAD patients with HFimpEF had fewer complications, and there were no statistically significant differences in terms of age and sex. Importantly, CAD patients with HFimpEF significantly achieved a 39% reduction in long-term mortality risk than patients with persistent HFrEF.

The burden of HF has increased to an estimated 23 million people globally, and approximately, 50% of cases are HFrEF, which results in millions of hospitalizations; hospitalized patients with HF continue to experience high post-discharge mortality and readmission rates (Ambrosy et al., 2014; Murphy et al., 2020). In addition, enhanced survival following AMI and the declining prevalence of hypertension and valvular heart disease are contributors to incident HF and have fueled the emergence of CAD as the primary risk factor for the development of HF (Lala and Desai, 2014). What complicates patient management is the difficulties clinicians face in terms of the uncertainties in the diagnosis of patients with CAD and HFrEF because of the non-specificity of the symptoms and signs of HF (Yancy et al., 2013). The evaluation of LVEF is among the most important and commonly used parameters in the diagnosis, characterization, prognosis, patient management, and treatment selection of HF (Lund et al., 2018). Improvement of left ventricular function is the one of the most important goals of HF therapy associated with reduced EF from any cause (Basuray et al., 2014).

It has been recently recognized that improvement of LVEF occurs in a proportion of HFrEF and is associated with a better prognosis. Multiple prior studies have documented outcomes with improved LVEF. In the study by Jørgensen et al. (2018) patients with improved LVEF (≥5% improvement) had a significantly lower risk of mortality and cardiovascular events compared with patients with persistently reduced LVEF. Perry et al. (2020) determined that an improvement in LVEF of ≥ 10% was independently associated with reduced mortality. Ghimire et al. (2019) also reported that HF patients with EF recovery (defined by EF absolute improvement≥10%) achieved a substantially better prognosis compared to patients with persistent HFrEF, even after multivariable adjustment. Taken together, these studies used different definitions for improvement or recovered LVEF.

The exact mechanisms underlying the association between CAD patients with HFimpEF and better prognosis have been extensively studied; hence, several hypotheses have been proposed. Howlett et al. (2020) indicated additional late improvement in LVEF and unchanged troponin levels for patients with HFimpEF, in contrast to those with persistent HFrEF and increase in serum troponin levels over time. These data provide evidence on the underlying mechanisms associated with late LV remodeling (Howlett et al., 2020). A large proportion of evidence supports reverse LV remodeling and recovery of LV function after implementation of evidence-based medical, device-based, and surgical interventions in patients with chronic HFrEF. Importantly, reverse LV remodeling is associated with improved myocyte contractility and improved LV chamber contractility (Wilcox et al., 2020). It is important to recognize that therapy-induced changes in LV remodeling are associated with fewer HF hospitalizations and decreased cardiovascular mortality and that there is a direct correlation between the extent of reverse LV remodeling and improvements in cardiac survival (Kramer et al., 2010). Importantly, in our study, we found that patients with CAD of younger age, with fewer comorbidities including PCI, history of PCI or AMI, and smaller left ventricular end diastolic diameter are more likely to advance to improved LVEF. Currently, there are no firm recommendations or guidelines on the role of PCI in managing HFrEF due to a lack of evidence from randomized controlled trials. The 2017 appropriateness criteria for revascularization do not provide a recommendation for PCI in patients with LVSD due to insufficient data (Parikh et al., 2021). At present, it is still controversial whether PCI is beneficial to left ventricular remodeling in patients with left ventricular dysfunction. In addition, the worse results in PCI patients could be explained by a more serious disease in patients with PCI. In addition, we noticed that neurohormonal medication that is proven to increase LVEF (especially beta-blockers) did not significantly alter our model. One reason was that our drug information was discharge medication. It cannot truly reflect whether patients take these drugs regularly and benefit from them. Further research is needed to validate these hypotheses and to explore the underlying mechanisms.

Risk stratification helps clinicians to screen patients with CAD whose EF may be difficult to improve in clinical practice and require investment of more medical resources. In fact, there is a substantial cross-over between treatment strategies for CAD and HFrEF, such as the use of β-blockers and an angiotensin receptor-neprilysin inhibitor, angiotensin-converting enzyme inhibitor, or angiotensin receptor blocker as foundational therapy, with addition of a mineralocorticoid receptor antagonist in patients with persistent symptoms (Chang et al., 2018). Ivabradine and hydralazine/isosorbide dinitrate also play a role in the care of certain patients with HFrEF (Murphy et al., 2020). More recently, sodium-glucose cotransporter 2 inhibitors have further improved disease outcomes and have reduced HF hospitalization in high-risk patients with HFrEF (Murphy et al., 2020). This may be an important treatment options for CAD patients with HFrEF in the future. In addition, EF is a poor index of myocardial efficacy and can be improved by the development of severe mitral regurgitation (MR) (Asgar et al., 2015; Goliasch et al., 2018). Thus, it is important to assess the severity of MR among CAD patients with HFrEF to guide and support clinical decisions and to establish treatment guidelines (Wilcox et al., 2020).

In addition, we found that patients with baseline LVEF and follow-up LVEF comprised a higher proportion of younger men, albeit there were no significant differences in terms of complications. Some drugs were used more frequently, and the mortality rates were lower, which was an interesting result. Since we recall patients for follow-up, younger men may pay closer attention to their health and may be suitable candidates for targeted drug therapy, thereby reducing mortality. However, this requires additional real-world data to explore the underlying relationship.

This study examined, for the first time, the characteristics and outcomes of the new definition of HFimpEF among patients with CAD. Although there are several limitations to be considered, the sizeable amount of data extracted from medical records allowed us to control a variety of confounders and selection bias in our analyses. First, the data were extracted from a single center, which hampered our control over a variety of confounders in analyses. Second, the population of our study may not represent all CAD patients with HFrEF because some patients without follow-up echocardiography were excluded. Third, we used echocardiography data from the 1-year follow-up to determine HFimpEF, even though we performed the sensitivity analysis and only included data from 6 months or more for the LVEF follow-up. Although the results did not change significantly, our sample size was too small to allow presenting them as main results. Fourth, we lack information about whether patients had accepted coronary artery bypass graft (CABG), which made it difficult to assess the role of CABG in our study. Finally, although we acquired information of LVEF of patients from very skilled cardiologists, LVEF data were determined without double-blinding, meaning that the two independent sonographers blinded from one another LVEF evaluation did not evaluate the LVEF of other. In addition, it is unknown how often biplane method of Simpson was used. This may lead to some errors in evaluating the improvement of ejection fraction. Finally, some potential confounding variables, such as drug compliance, valve disease, complete revascularization, and on the history of HF, were not considered in this study. However, our data analysis included the main clinical parameters.

## Conclusion

Our study demonstrated that HFimpEF significantly reduced long-term mortality risk by 39% compared to patients with persistent HFrEF, which may serve as an effective end point evaluable in future studies. In addition, many independent important risk predictors such as younger age, fewer clinical comorbidities, and lower left ventricular end diastolic diameter are identified, and these predictors may be used to optimize patient care among individuals with persistent HFrEF. Importantly, more prospective studies, including randomized controlled trials, are needed to improve the recognition of the new definition of HF, which is more conducive to accurate diagnosis and treatment indications among patients with CAD.

## Data Availability Statement

The data analyzed in this study is subject to the following licenses/restrictions: Not applicable. Requests to access these datasets should be directed to YL, liuyong@gdph.org.cn.

## Ethics Statement

The studies involving human participants were reviewed and approved by the Guangdong Provincial People’s Hospital Ethics Committee. Written informed consent for participation was not required for this study in accordance with the national legislation and the institutional requirements.

## Author Contributions

YL, NT, JC, YHu, and C-QO designed the study. SC, YHe, and BW collected and reviewed clinical and laboratory data. SC, HH, QL, and WL analyzed data. SC, HH, YHe, and BW performed the statistical analysis. HH, JL, ML, ZY, and KB performed drafting or revision of the manuscript. YL, NT, MA, and JC reviewed, interpreted, and checked clinical data. All authors contributed to the article and approved the submitted version.

## Conflict of Interest

The authors declare that the research was conducted in the absence of any commercial or financial relationships that could be construed as a potential conflict of interest.

## Publisher’s Note

All claims expressed in this article are solely those of the authors and do not necessarily represent those of their affiliated organizations, or those of the publisher, the editors and the reviewers. Any product that may be evaluated in this article, or claim that may be made by its manufacturer, is not guaranteed or endorsed by the publisher.
