# Supplementary material for: A Universal New Definition of Heart Failure With Improved Ejection Fraction for Patients With Coronary Artery Disease
Source: Front Physiol. 2021 Dec 3;12:770650. doi: 10.3389/fphys.2021.770650 (PMC8678467; doi:10.3389/fphys.2021.770650)
Supplement: Supplementary file 1 [file Table_1.DOCX]

**Supplement Table 1 Sensitivity analysis of data filtering process.**

| **Characteristic** | **Overall** | **Patients without LVEF follow up** | **Patients with LVEF follow up** | **P-value** |
| --- | --- | --- | --- | --- |
|  | (n=3441) | (n=2694) | (n=747) |  |
| **Demographic characteristics** | | | | |
| Male, n (%) | 2859 (83.1) | 2211 (82.1) | 648 (86.7) | 0.003 |
| Age, years, mean (SD) | 63.7 (10.76) | 64.3 (10.6) | 61.4 (11.0) | <0.001 |
| Medical insurance |  |  |  |  |
| Self-paying | 523 (15.2) | 430 (16.0) | 93 (12.4) | <0.001 |
| Urban insurance | 2378 (69.1) | 1812 (67.3) | 566 (75.8) |  |
| Rural insurance | 90 (2.6) | 63 (2.3) | 27 (3.6) |  |
| Other | 449 (13.1) | 388 (14.4) | 61 (8.2) |  |
| **Medical history** | | | | |
| AMI, n (%) | 883 (25.7) | 710 (26.4) | 173 (23.2) | 0.084 |
| DM, n(%) | 1189 (34.6) | 926 (34.4) | 263 (35.2) | 0.708 |
| CKD, n(%) | 1205 (35.0) | 946 (35.1) | 259 (34.7) | 0.856 |
| AF, n (%) | 160 (4.6) | 129 (4.8) | 31 (4.1) | 0.525 |
| Stroke, n (%) | 238 (6.9) | 197 (7.3) | 41 (5.5) | 0.097 |
| History of cancer, n (%) | 31 (0.9) | 23 (0.9) | 8 (1.1) | 0.737 |
| COPD, n (%) | 44 (1.3) | 38 (1.4) | 6 (0.8) | 0.261 |
| Hypertension, n (%) | 1676 (48.7) | 1317 (48.9) | 359 (48.1) | 0.713 |
| History of AMI, n (%) | 506 (14.7) | 414 (15.4) | 92 (12.3) | 0.042 |
| History of CABG, n (%) | 43 (1.2) | 36 (1.3) | 7 (0.9) | 0.494 |
| History of prePCI, n (%) | 423 (12.3) | 338 (12.6) | 85 (11.4) | 0.424 |
| **Procedure, n (%)** | | | | |
| PCI, n (%) | 2482 (72.1) | 1932 (71.7) | 550 (73.6) | 0.324 |
| DES, n (%) | 2337 (67.9) | 1812 (67.3) | 525 (70.3) | 0.128 |
| BES, n (%) | 86 (2.5) | 69 (2.6) | 17 (2.3) | 0.757 |
| CMV (mean (SD)) | 146.0 (85.6) | 145.6 (84.7) | 147.6 (88.7) | 0.571 |
| **Laboratory tests** | | | | |
| eGFR (mean (SD)) | 66.1 (25.2) | 65.6 (25.2) | 67.7 (25.1) | 0.065 |
| HDLC (median [IQR]) | 0.92 [0.77, 1.10] | 0.92 [0.77, 1.11] | 0.91 [0.77, 1.08] | 0.065 |
| ALB (mean (SD)) | 34.5 (4.6) | 34.3 (4.6) | 35.3 (4.5) | <0.001 |
| HS_CRP (median [IQR]) | 7.09 [2.39, 20.20] | 6.92 [2.25, 19.40] | 7.36 [2.62, 20.90] | 0.4 |
| LVEDD, mm (mean (SD)) | 59.4(12.6) | 59.0(8.2) | 60.7(22.0) | 0.001 |
| **Medications** | | | | |
| Beta blocker, n (%) | 2791 (84.2) | 2150 (83.4) | 641 (87.0) | 0.022 |
| Statins, n(%) | 3069 (92.6) | 2370 (91.9) | 699 (94.8) | 0.01 |
| Aspirin, n(%) | 2955 (89.1) | 2300 (89.2) | 655 (88.9) | 0.844 |
| Clopidogrel, n (%) | 2742 (82.7) | 2122 (82.3) | 620 (84.1) | 0.275 |
| CCB, n (%) | 368 (11.1) | 279 (10.8) | 89 (12.1) | 0.374 |
| ACEI/ARB, n (%) | 1804 (54.4) | 1404 (54.5) | 400 (54.3) | 0.962 |
| Spirolactone, n (%) | 1924 (58.0) | 1421 (55.1) | 503 (68.2) | <0.001 |
| Discharge status, n (%) |  |  |  |  |
| Medical advice discharge | 29 (0.8) | 28 (1.0) | 1 (0.1) | <0.001 |
| Automatic withdraw | 52 (1.5) | 50 (1.9) | 2 (0.3) |  |
| In-hospital death | 14 (0.4) | 14 (0.5) | 0 (0.0) |  |
| **Long-term mortality** | 827 (24.0) | 692 (25.7) | 135 (18.1) | <0.001 |

ACEI/ARB = angiotensin-converting enzyme inhibitor/angiotensin receptor blocker; AF, atrial fibrillation; ALB, albumin ; AMI, acute myocardial infarction; BES, bare metal stent; CABG, Coronary Artery Bypass Grafting; CCB = calcium channel blockers;CKD, chronic kidney diseases ; COPD,chronic obstructive pulmonary disease; DES, drug eluting stents; DM, diabetes mellitus ; eGFR = estimated glomerular filtration rate; HbA1c = hemoglobin A1c; HDL-C= hight-density lipoprotein cholesterol; hs-CRP = hypersensitive C-reactive protein; PCI = percutaneous coronary intervention;  LDL-C=low-density lipoprotein cholesterol; MRA = aldosterone receptor antagonist
