# Supplementary material for: A Universal New Definition of Heart Failure With Improved Ejection Fraction for Patients With Coronary Artery Disease
Source: Front Physiol. 2021 Dec 3;12:770650. doi: 10.3389/fphys.2021.770650 (PMC8678467; doi:10.3389/fphys.2021.770650)
Supplement: Supplementary file 2 [file Table_2.DOCX]

**Supplement Table 2** Sensitivity analysis of multiple fill analysis.

| Predictors for HFimpEF among baseline LVEF≤40 | | |
| --- | --- | --- |
|  | OR（95%Cl） | aOR（95%Cl）^*^ |
| Age, per 1 year increase | 0.99 (0.98-1.01) | 0.98(0.97-0.99) |
| Female (vs male) | 1.17(0.75-1.80) |  |
| CKD (vs Non-CKD) | 0.74 (0.54-1.01) | 0.71 (0.50-1.00) |
| AF (vs Non-AF) | 1.15 (0.54-2.39) |  |
| COPD (vs Non-COPD) | 0.90 (0.12-4.66) |  |
| PCI (vs Non-PCI) | 0.60 (0.42-0.84) | 0.47(0.33-0.68) |
| History of AMI (Yes vs No) | 0.32(0.17-0.55) | 0.37(0.19-0.65) |
| History of PCI (Yes vs No) | 0.44(0.24-0.74) | 0.46(0.25-0.80) |
| hypertension | 1.33(0.98-1.81) | 1.39 (1.00-1.93) |
| Baseline ejection fraction, per 1% increase | 1.03(1.01-1.06) |  |
| Left ventricular end diastolic diameter, per 1mm increase | 0.93(0.91-0.95) | 0.92(0.90-0.94) |
| Final Model C-index = 0.71 |  |  |

**^*^ Backward stepwise logistic regression**

**Abbreviation:** CKD, chronic kidney diseases; AF, atrial fibrillation; COPD, chronic obstructive pulmonary disease; AMI, acute myocardial infarction; PCI, percutaneous coronary intervention
