# Supplementary material for: A Universal New Definition of Heart Failure With Improved Ejection Fraction for Patients With Coronary Artery Disease
Source: Front Physiol. 2021 Dec 3;12:770650. doi: 10.3389/fphys.2021.770650 (PMC8678467; doi:10.3389/fphys.2021.770650)
Supplement: Supplementary file 3 [file Table_3.DOCX]

**Supplement Table 3** The association between HFrEF and mortality in different models among CAD patients (6 months or more of LVEF follow-up).

|  |  | Long-term all-cause mortality | |
| --- | --- | --- | --- |
|  | Death/Total | HR, 95%Cl, P-value | |
|  |  | Model 1^a^ | Model 2^b^ |
| Persistent HFrEF | 34/172 | ref | ref |
| HFimpEF | 12/118 | 0.51(0.27-0.99), 0.04 | 0.49(0.25-0.95), 0.04 |

^a^, unadjusted;^b^, adjusted age and gender
